# Investigation on L-rhamnose metabolism of *Loigolactobacillus coryniformis* subsp. *coryniformis* DSM 20001 and its propionate-containing fermentates

**DOI:** 10.1128/aem.01613-24

**Published:** 2024-12-18

**Authors:** Mensure Elvan Gezer, Kathrine Gravlund Fønss, Maria Florencia Bambace, Angeliki Marietou, Sanne Sandberg Overby, Ulrik Sundekilde, Clarissa Schwab

**Affiliations:** 1 Department of Biological and Chemical Engineering, Aarhus University 1006 , Aarhus, Denmark; 2 Department of Food Science, Aarhus University 1006 , Aarhus, Denmark; INRS Armand-Frappier Sante Biotechnologie Research Centre, Laval, Canada

**Keywords:** biopreservation, *Loigolactobacillus coryniformis*, L-rhamnose, 1,2-propanediol

## Abstract

Propionate is an important short-chain carboxylic acid (SCCA) that serves as an effective antimicrobial agent for food preservation. Previous research has highlighted that few *Lactobacillaceae* can synthesize propionate by metabolizing deoxyhexoses via the fermentation intermediate 1,2-propanediol (1,2-PD). In this study, we investigated propionate production by *Loigolactobacillus coryniformis* subsp. *coryniformis* DSM 20001 while utilizing L-rhamnose as the primary carbon source. We cultivated *L. coryniformis* in small-scale anaerobic bioreactors at 30°C and pH 6.5 for 72 h and monitored the expression of key genes associated with deoxyhexose metabolism using quantitative PCR. In addition, we assessed the contribution of individual SCCA to the antimicrobial activity of the fermentate against common foodborne pathogens, including *Escherichia coli*, *Salmonella enterica*, *Klebsiella oxytoca*, *Staphylococcus aureus*, *Candida albicans*, *Aspergillus niger*, *Penicillium roqueforti*, and *Penicillium purpurogenum* using broth dilution assays. During cultivation, we observed the production of up to 16 mM propionate, alongside other metabolites such as lactate (26 mM), formate (2 mM), and acetate (4 mM), derived from 32 mM L-rhamnose. Genes related to L-rhamnose utilization were upregulated within the initial 48 h, while genes involved in 1,2-PD utilization remained highly transcribed throughout fermentation. Comparing the antimicrobial efficacy of the fermentates to synthetic SCCA mixtures, bacterial indicator strains were more sensitive than molds and yeast. Propionate was the primary SCCA responsible for inhibitory activity; inhibition was reduced if indicator strains were able to use lactate.

**IMPORTANCE:**

Worldwide, approximately 30% of food produced is lost. Despite the application of complementary treatment methods, microbial food spoilage can occur along the entire value chain. The rising concern about food waste has led to increasing interest in natural preservation approaches. *Lactobacillaceae* fermentative systems produce a variety of short-chain carboxylic acid (SCCA) with antimicrobial potential, and we present here fundamental insight into the only recently discovered deoxyhexose metabolism of *Loigolactobacillus coryniformis* producing the antimicrobial SCCA propionate. We developed a bioprocess to produce propionate from L-rhamnose under controlled conditions as a first step toward the exploitation of L-rhamnose metabolism in the production of antimicrobial fermentates for use in the food industry, potentially replacing chemical alternatives. Our investigations highlight the major contribution of propionate in antimicrobial activity but also indicate the issue of co-occurring fermentable metabolites, which can affect the efficiency of fermentates.

## INTRODUCTION

Propionate is a naturally occurring short-chain carboxylic acid (SCCA) used as a food preservative suppressing the growth of bacterial and fungal spoilage and pathogenic microorganisms. The biological activity of SCCA is influenced by acid strength (pKa). The weak acid theory suggests that undissociated (more hydrophobic) acids are more likely to cross the cell membrane (1). With a pKa of 3.86, lactic acid, the major SCCA associated with the biopreservation of fermented foods, is a stronger acid but weaker antimicrobial than propionate. The antimicrobial activity of weak acids is higher in an environment with a low pH, and propionic acid (pKa value is 4.87) is higher in its undissociated form at pH 4.5 (1).

*Lactobacillaceae* produce SCCA through carbohydrate fermentation. During homofermentative metabolism, 1 M glucose theoretically yields 2 M lactate as the main fermentation product. When substrate availability is limited, acetate, formate, and ethanol accumulate as products of pyruvate metabolism (2, 3). An important enzyme is pyruvate formate lyase that is inhibited by glucose (4, 5). Heterofermentative *Lactobacillaceae* metabolize 1 M glucose to 1 M lactate, 1 M ethanol, and 1 M CO_2_ and prefer sucrose or maltose due to higher energy yield during utilization (3). The formation of propionate has been reported for consortia of the silage species *Lentilactobacillus buchneri* and *Lentilactobacillus diolivorans*, which together metabolize lactate to 1,2-propanediol (1,2-PD) and 1,2-PD to propionate (6). Recently, synthetic consortia of *Lactobacillaceae* were shown to form propionate from deoxyhexoses (7). For example, *Lacticaseibacillus rhamnosus* produced 1,2-PD from L-rhamnose or L-fucose (7, 8), which was transformed into propanal, propionate, and propanol by strains of *Limosilactobacillus reuteri* (7, 9).

**Fig 1:**
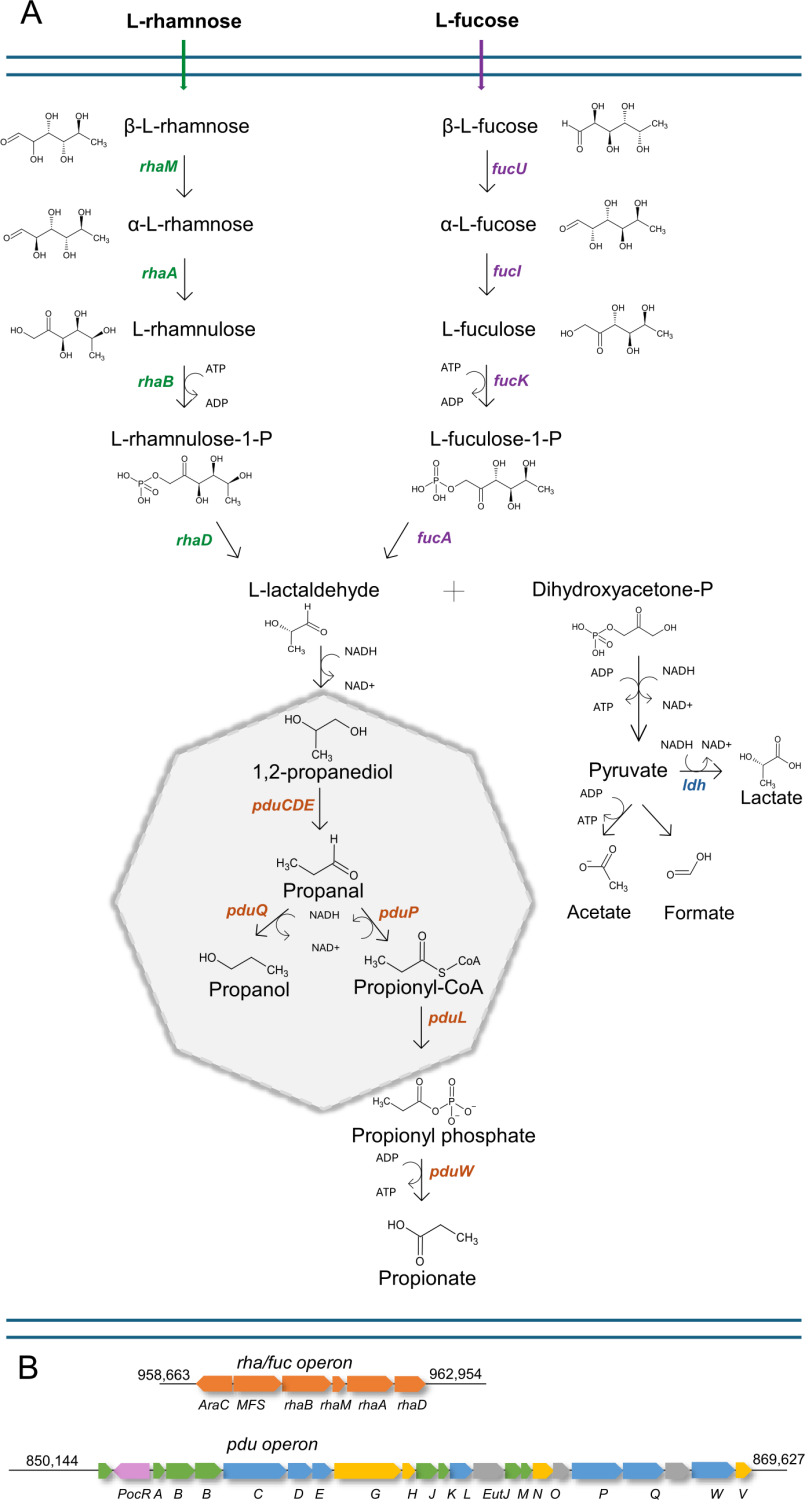
Proposed metabolic pathway of L-rhamnose catabolism. (**A**). L-rhamnose and L-fucose utilization in *Loigolactobacillus coryniformis* subsp. *coryniformis* DSM 20001 (GCF_002706425.1) follows the same pathway as described in *L. rhamnosus* (8). The proposed pathway of cobalamin-dependent 1,2-PD metabolism in *L. coryniformis* is based on the genome and is analogous to *L. reuteri* DSM 20016, which partly occurs in a bacterial microcompartment (grey) (16). All the genes specifying the required enzymes were found in the *L. coryniformis* (DSM 20001) *pdu* operon (**B**). Gene clusters encoding proteins related to L-rhamnose, L-fucose and 1,2-PD utilization. Gene abbreviations: *rhaM*, L-rhamnose mutarotase; *fucU*, L-fucose mutarotase; *rhaA*, L-rhamnose isomerase; *fucI*, L-fucose isomerase; *rhaB*, rhamnulokinase; *fucK*, L-fuculose kinase; *rhaD*, rhamnulose-1-phosphate aldolase; *fucA*, L-fuculose-1-phosphate aldolase; *ldh*, L-lactate dehydrogenase; *pduC*, propanediol/glycerol family dehydratase large subunit; *pduD*, propanediol/glycerol family dehydratase medium subunit; *pduE*, diol dehydratase small subunit; *pduG*, diol dehydratase reactivase subunit alpha; *pduH*, glycerol dehydratase reactivase beta/small subunit family protein; *pduJ,* BMC domain-containing protein; *pduK*, BMC domain-containing protein; *pduL*, phosphate propanoyltransferase; *pduM*, PduM family microcompartment protein; *pduN*, EutN/CcmL family microcompartment protein; *pduO*, Cob(l)yrinic acid, a,c-diamide adenosyltransferase; *pduP*, aldehyde dehydrogenase family protein; *pduQ*, 1-propanol dehydrogenase; *pduW,* acetate kinase; *pduV*, EutP/pduV family microcompartment system protein.

L-rhamnose conversion to propionate by a single strain of *Lactobacillaceae* has been only documented for *Loigolactobacillus coryniformis* subsp. *coryniformis* DSM 20001 (7). *L. coryniformis* produced 1,2-PD (2.1 mM) and low levels (2.5 ± 1.3 mM) of propionate from around 50 mM L-rhamnose (7). Based on its genome, *L. coryniformis* DSM 20001 (GCF_002706425.1) possesses the genes transcribing proteins associated with L-rhamnose/L-fucose metabolism and harbors the *pdu* operon related to 1,2-PD metabolism (Fig. 1). In general, L-rhamnose or L-fucose is transported into the cells via a specific ATP-binding cassette (ABC) (10) and/or major facilitator superfamily (MFS) transporter systems (11). L-rhamnose mutarotase (RhaM) (or its L-fucose utilizing analog L-fucose mutarotase [FucU]), L-rhamnose isomerase (RhaA) (L-fucose isomerase [FucI]), rhamnulosekinase (RhaB) (L-fuculose kinase [FucK]), and rhamnulose-1-phosphate aldolase (RhaD) (L-fuculose-1-phosphate aldolase [FucA]) transform L-rhamnose or L-fucose into L-lactaldehyde and dihydroxyacetone phosphate (DHAP), which serves as an intermediate for pyruvate metabolism (8, 12). L-lactaldehyde is oxidoreduced to 1,2-PD by 1,2-propanediol oxidoreductase (FucO), followed by further metabolism to propionate by genes encoded by the *pdu* operon that includes the cobalamin-dependent glycerol/diol dehydratase PduCDE (13, 14). The resulting propanal can be metabolized to propionate by phosphate propionaldehyde dehydrogenase (PduP), propanoyltransferase (PduL), and propionate kinase (PduW) and/or to propanol by alcohol dehydrogenase (PduQ) (15). Due to the toxicity of propanal, part of the 1,2-PD transformation occurs in a bacterial microcompartment (BMC) (Fig. 1).

There is increasing current interest in utilizing natural fermentation systems for biopreservation. Deoxyhexose metabolism by *L. coryniformis* allows to expand the portfolio of available food antimicrobials. With the overall goal to establish the potential of a newly described pathway of *L. coryniformis* for the natural preservation of food, this study combined three investigations: (i) optimization of propionate production from L-rhamnose by batch fermentation of *L. coryniformis* under pH- and temperature-controlled conditions, (ii) determination of expression profiles of genes related to deoxyhexose and 1,2-PD metabolism during optimal production, and (iii) examination of the antibacterial and antifungal activities of the resulting fermentates.

## MATERIALS AND METHODS

### Strains and culture conditions

*Loigolactobacillus coryniformis* subsp. *coryniformis* DSM 20001, *Escherichia coli* DSM 19206, *Klebsiella oxytoca* DSM 5115, *Staphylococcus aureus* DSM 1104, *Candida albicans* DSM 10697, and *Penicillium purpurogenum* DSM 21170 were purchased from the Deutsche Sammlung von Mikroorganismen und Zellkulturen GmbH (DSMZ). *Salmonella enterica* CCM 4420 was obtained from the Czech Collection of Microorganisms (CCM). *Pichia kluyveri* FMT 3000 and *Saccharomyces cerevisiae* FMT 3003 were provided by the culture collection of the Functional Microbe Technology (FMT) group at Aarhus University. *Penicillium roqueforti* (lot. no. 05110069) was purchased from Dansk Hjemmeproduction. *Aspergillus niger* BCE M1 was obtained from the mold collection of the Biological and Chemical Engineering Department at Aarhus University.

*L. coryniformis* was routinely cultivated in modified Wilkins-Chalgren anaerobe broth (WCSP) supplemented with soya peptone, L-cysteine, and Tween 80 at 30°C. WSCP was prepared anaerobically by boiling the medium followed by cooling under CO_2_ flow. When the temperature was approx. 60°C, L-cysteine HCl (0.5 g/L final concentration) was added. WCSP was distributed to Hungate tubes (10 mL) under constant CO_2_ flow, and tubes were closed and autoclaved.

To obtain working cultures, *L. coryniformis* glycerol stocks stored at −80°C were streaked on WCSP agar and incubated anaerobically at 30°C for 48 h using AnaeroGenTM 2.5-L sachets (Thermo Scientific). Bacteria (*E. coli*, *S. enterica*, *K. oxytoca*, and *S. aureus*) and yeast (*C. albicans*, *P. kluyveri*, and *S. cerevisiae*) indicator strains were streaked on Luria Bertani (LB) or yeast peptone dextrose (YPD) agar and incubated aerobically at 37°C for 24 h or at room temperature for 72 h, respectively. Single colonies were picked from agar plates and incubated in the respective broths for 24 h (or 48 h for yeasts), followed by a second cultivation with inoculation of 1% (vol/vol) before the test trials. Molds *A. niger*, *P. roqueforti*, and *P. purpurogenum* were grown on potato dextrose agar (PDA) at 25°C for 7 days. To harvest spores from PDA plates, mycelia were vigorously mixed with sterile distilled water using a sterile disposable cell spreader.

To determine CFU of bacteria and yeast, working cultures and bioreactor effluents were serially diluted in sterile peptone water and plated on the respective media. Plates for enumeration of *L. coryniformis* were incubated under anaerobic conditions at 30°C for 48 h, while *S. enterica*, *E. coli*, *S. aureus*, and *K. oxytoca* were counted after incubation at 37°C for 48 h. Yeasts were incubated at room temperature for 72 h. Colonies were counted after incubation, and cell counts were expressed as log CFU/mL. Spore concentrations were determined using a Thoma cell counting chamber (Marienfeld).

### Small-scale batch fermentations of *L. coryniformis*

Small-scale batch fermentations of *L. coryniformis* were carried out in bioreactors using the semi-defined yeast extract casitone (YC) medium as a growth medium (7). Briefly, YC was prepared containing amicase (10 g/L), L-cysteine ( 0.5 g/L), NaHCO_3_ (4 g/L), yeast extract (2.5 g/L), mineral solution (300 mL/L), MEM Vitamin Solution 100× (1 mL/L), and 5% of hemin solution (20 µL/L). As a carbon source, L-rhamnose (32 mM) was added to the medium, and D-glucose (2 mM) was added to support the initial growth (17).

We used a Lambda Minifor bioreactor system with 35–400-mL working capacity vessels. The working volume was 300 mL. The fermentations were maintained at 30°C, stirring 0.5 Hz, CO_2_ flow of 0.04 L/min, and pH 6.5 for 72 h. Samples were collected from the bioreactors at 0, 6, 24, 48, and 72 h of fermentation. Optical density was recorded directly after sample collection using a Cell Density Meter (Fisher Scientific) at 600 nm (OD_600_). CFU were determined as described above.

Effluent samples were centrifuged and stored as cell biomass and cell-free fermentates at −20°C until further analysis. Bioreactor fermentations were conducted in five independent replicates F1–F5. We collected biomass from fermentations 4 (F4) and 5 (F5), quick frozen in liquid nitrogen, and stored at −80°C for RNA extraction.

### DNA and RNA isolation, reverse transcription, and quantitative PCR

DNA isolation of *L. coryniformis* was performed using the GeneJet Genomic DNA Purification Kit according to the manufacturer’s instructions (Thermo Scientific) to determine the efficiencies of primers.

Total RNA was extracted from cell pellets collected at regular intervals in F4 and F5 fermentations using the RNeasy Plus Universal Mini Kit (Qiagen), including RNase-free DNase I treatment (Thermo Scientific). The quality of the RNA was examined by determining the 260/280 absorbance ratio using a NanoDrop (DeNovix DS-11, Thermo Fisher Scientific). To confirm the absence of chromosomal DNA, PCR was run using primers 27F (AGAGTTTGATCCTGGCTCAG) and 1492R (GGTTACCTTGTTACGACTT). The PCR was performed in a total volume of 25 µL. Each reaction contained 2.5 µL of green PCR buffer (Invitrogen), 2.5 µL of dNTPs (Thermo Scientific), 2.5 µL of DreamTaq polymerase (Thermo Scientific), 1 µL of each of the forward and reverse primers (10 µM), 1 µL of template DNA, and 16.8 µL of nuclease-free water. The reaction conditions on the Eppendorf Mastercycler nexus GX2 thermocycler were as follows: initial denaturation at 95°C for 10 min; 30 cycles of denaturation at 95°C for 30 s, annealing at 52°C for 30 s, and extension at 72°C for 2 min; and a final extension at 72°C for 3 min. Agarose gel (1%) electrophoresis was carried out to verify the absence of amplicons. Purified RNA was used for cDNA synthesis with the Superscript III First Strand kit (Invitrogen) following the manufacturer’s instructions.

Four genes related to *L. coryniformis* L-rhamnose metabolism were selected to determine L-rhamnose-dependent gene expression, *rhaA*, *rhaM*, *rhaB*, and *rhaD*, in addition to *pduC* and *pduL*, which are transcribed by the *pdu* operon, and *ldh* that is related to lactate formation. As housekeeping genes, this study used *gap*, *gyrB*, *pheS*, and 16S rRNA genes. Gene sequences were retrieved from the National Center of Biotechnology Information (GCF_002706425.1). The primers were designed by the Eurofins Genomics PCR Primer Design Tool (https://eurofinsgenomics.eu/en/ecom/tools/pcr-primer-design/) (Table 1).

**TABLE 1:** Target genes and primers used in quantitative PCR (qPCR) reactions^*a*^

	Target gene	Sequence (5'-3')	Length (bp)	Efficiency (%)
L-rhamnose metabolism-related genes	L-rhamnose mutarotase (*rhaM*) (Gene ID: 65916556)	F: 5′-AGGAACATGGGGCTACCAAC-3′ R: 5′-AAGATCATCGGCTACCGGAC-3′	191	92
	L-rhamnose isomerase (*rhaA*) (Gene ID: 65916557)	F: 5′-GGGACAGGGATCGAATCTTAC-3′ R: 5´- CAACGAACAGGACGAGAAAC −3`	188	91
	Rhamnulokinase (*rhaB*) (Gene ID: 65916555)	F: 5′-TTACGACCGCGACCCATGATAC-3′ R: 5′-TCACGAAAAGCAGCCAACCC-3′	145	88
	Rhamnulose-1-phosphate aldolase (*rhaD*) (Gene ID: 65916558)	F: 5´- GACGAAATGCGTAAAATCACC −3` R: 5´- AACATTCTTGAAGTAGCGACC −3`	133	89
	L-lactate dehydrogenase (*ldh*) (Gene ID: 65918298)	F: 5′-ACACGCAAATTAGCAGTTATCG-3′ R: 5′-GCATCAGCAGCCACTTTTTC-3′	128	96
1,2-PD metabolism-related genes	Cobalamin-dependent glycerol/diol dehydratases (*pduC*) (Gene ID: 65916455)	F: 5′-CGTTATGCACCATTCAATGCT-3′ R: 5′-CCATGGAGTATCATCACCATC-3′		99
	Phosphate propanoyltransferase (*pduL*) (Gene ID: 65916462)	F: 5′-GTTTCTAACCGCCATGTCC-3′ R: 5′-CCGAACGAGCTAATTCAACC-3′	211	96
Housekeeping genes	Type I glyceraldehyde-3-phosphate dehydrogenase (*gap*) (Gene ID: 65917734)	F: 5′-TCCAAAGCTAAATCACAAGCTC-3′ R: 5′-CCAACTTCAACGCCAAATTC-3′	212	96
	DNA topoisomerase (ATP-hydrolyzing) subunit B (*gyrB*) (Gene ID: 65915625)	F: 5′-GCCCGATCCAGATATTTTCC-3′ R: 5′-CCCTTTTTCAACACCTTCAAC-3′	244	92
	Phenylalanine-tRNA ligase subunit alpha (*pheS*) (Gene ID: 65917333)	F: 5′-ACCCATTCACATCAATTCTTCC-3′ R: 5′-CATACCCGCACCTAATACTTC-3′	255	93
	16S rRNA gene	F: 5′-ACTCCTACGGGAGGCAGCAG-3′ R: 5′-ATTACCGCGGCTGCTGG-3′		112

^*a*^ Primers were constructed based on the genome of *L. coryniformis* subsp. *coryniformis* DSM 20001. Four housekeeping genes (*gap*, *gyrB*, *pheS*, and 16S rRNA) were chosen to investigate the relative expression levels of genes responsible for L-rhamnose and 1,2-PD metabolisms.

To generate amplicons for standard curves and to determine primer efficiency, PCR analysis was performed using genomic DNA (Mastercycler nexus GX2). PCR products were cleaned using the Monarch PCR & DNA Cleanup Kit (New England Biolabs) and serially diluted with nuclease-free water.

Quantitative PCR (qPCR) reactions were performed with a CFX Connect Real-Time System using iTaq Universal SYBR Green Supermix (Bio-Rad Laboratories) using purified PCR amplicons or cDNA as a template. Each reaction consisted of 2 µL nuclease-free water, 5 µL iTaq Supermix, 1 µL of each primer, and 1 µL of cDNA or diluted PCR amplicons. Samples and blanks were run in duplicate on the same plate for 40 cycles (3 min at 95°C, 10 s at 95°C, and 30 s at 60°C). Reactions were completed within 10 s at 95°C and finally a melting curve from 65°C to 95°C with an increment of 0.5°C for 5 s. Two biological replicates were analyzed for each sample.

The efficiencies of primers were calculated using the following equation:


$$Converted\;primer\;efficiency=\;\left(\frac{primer\;efficiency\;\left(\%\right)}{100}\right)+1$$

To determine relative expression levels, Ct values were obtained for samples collected at 6, 24, 48, and 72 h for target (genes of interest) and housekeeping genes. The average Ct values of technical replicates were calculated for all samples. Expression at 6 h of fermentation was chosen as a calibrator/reference and was used to calculate the ∆Ct values for all samples and all genes using the following equation.


ΔCt= (Ct expression 6 h incubation)− (Ct expression n h incubation)

After determining the ∆Ct values and efficiencies of all tested genes, the following equation was used to calculate the gene expression ratio of each sample.


$$Gene\;expression\;ratio=\frac{{\left(efficiency\;of\;genes\;of\;interest\right)}^{\Delta Ct\;genes\;of\;interest}}{{\left(efficiency\;of\;housekeeping\;genes\right)}^{\Delta Ct\;housekeeping\;genes}}$$

### High-performance liquid chromatography with refractive index detector (HPLC-RI)

The levels of substrates and fermentation metabolites in bioreactor-derived, cell-free fermentates and synthetic SCCA (sSCCA) preparations were determined using a 1260 Infinity II LC with refractive index (RI) detection (Agilent Technologies). The samples (10 µL) were separated on a Hi-Plex H guard and main column (300-mm × 7.7-mm internal diameter × 8-µm particle size, Agilent Technologies) and eluted with 5 mM H_2_SO_4_ as the mobile phase at a flow rate of 0.6 mL min^−1^ at 40°C. Glucose, L-rhamnose, and fermentation metabolites were quantified using external standards.

### ^1^H-Nuclear magnetic resonance spectroscopy

We used ^1^H-nuclear magnetic resonance spectroscopy (^1^H-NMR) spectrometry to verify the production of propionate and other SCCA in bioreactor fermentations as described (7). Briefly, 400 µL of the cell-free fermentate samples mixed with 200 µL D_2_O and 0.32 mM trimethylsilylpropanoic acid (TSP) were transferred into 5-mm NMR tubes. ^1^H-NMR spectroscopy was performed using a Bruker NEO-IVDR 600 NMR spectrometer, operating at a ^1^H frequency of 600.03 MHz and with a 5-mm ^1^H BBI probe (Bruker BioSpin). TSP was used as an internal chemical shift reference and quantification standard, and all ^1^H spectra were referenced to the TSP signal at 0 ppm. Manual phase and baseline spectra were corrected using Topspin 4.09 (Bruker BioSpin). Signals were assigned and quantified using Chenomx NMR Suite 8.6 (Chenomx Inc.).

### Preparation of synthetic SCCA (sSCCA) mixtures

Stock solutions (100 mM) of acetate, lactate, propionate, and formate were prepared in pH-adjusted autoclaved LB (pH 4.5 and 5.5), YPD (pH 4.5), and WCSP (pH 4.5). Levels of acetate, lactate, propionate, and formate present in the fermentates, as determined by HPLC-RI, were used to prepare sSCCA mixtures. In addition, sSCCA mixtures were prepared without acetate (sSCCA-A), lactate (sSCCA-L), or propionate (sSCCA-P) to identify the inhibitory contribution of the individual SCCAs. All sSCCA mixtures were filter sterilized (0.2-µm syringe filter).

### Antimicrobial activity assays

The antimicrobial activity of individual SCCA, cell-free fermentates, and sSCCA mixtures was determined using broth dilution assays. The pH of the growth medium and the fermentates was adjusted to 4.5 for all strains except *S. aureus* (pH 5.5). A twofold dilution series was conducted using a liquid handling robot (Gilson PipetMax). The final volume in each well was 100 µL. Wells were inoculated with 10 µL of the indicator strain suspension. The starting cell counts of *E. coli*, *K. oxytoca*, and *S. enterica* were 5.9 log CFU/mL, 6.7 log CFU/mL, and 6.5 log CFU/mL, respectively. *S. aureus* was added to obtain counts of 6.4 log CFU/mL. The cell count of *C. albicans* was 3.5 log CFU/mL, and 3 log CFU/mL for *S. cerevisiae* and *P. kluyveri*. Mold spore levels were adjusted to 10^4^ spores/mL of WCSP broth before inoculation. Positive (cultivation broth and cell suspensions of indicator strains) and negative (only broth) controls were included in each assay. Microtiter plates were incubated at 37°C for 24 h (bacteria) or at 25°C for 48 h (yeast and molds). After incubation, optical density was recorded at 600 nm using a microplate reader (Infinite M200 Pro, Tecan). For molds, the inhibitory concentrations were determined visually. Technical duplicates were performed in each assay for the tested inhibitors except for fermentates; bacteria and fungi were tested in four biological replicates and molds in two biological replicates.

The OD_600_ values obtained from antimicrobial activity assays were analyzed using a four-parameter logistic regression (a sigmoidal curve) using GraphPad Prism 9 software. This analysis allowed us to calculate the minimum inhibitory concentration that reduced density to 50% (MIC_50_) of individual SCCA and to determine the dilution factor required for the fermentates and sSCCA mixtures to reduce cell density to 50% in broth dilution assays (DF_50_). For molds, we recorded reduced growth visually and defined the minimal inhibitory concentration as the concentration that inhibited the outgrowth of the spores completely (MIC). Concentrations with reduced growth compared to positive controls were used to calculate the DF.

### Statistical analysis

The MIC_50_ values for the individual SCCAs within the indicator strains were compared by one-way analysis of variance (ANOVA) with Tukey’s test for multiple comparisons conducted using R-Studio software. The DF_50_ values for each replicate were compared within the indicator strain by ANOVA with Tukey’s test. A Shapiro-Wilk test was performed before ANOVA using R-Studio software to ensure a normal data distribution. The value of *P* < 0.05 was considered significant for all statistical analyses.

## RESULTS

### Metabolite production from L-rhamnose by *L. coryniformis*

We conducted a small-scale batch fermentation to explore propionate production from L-rhamnose metabolism by *L. coryniformis*. The fermentation medium consisted of YC broth supplemented with L-rhamnose (32 ± 6 mM). D-glucose (1.2 ± 1.1 mM) was added to support initial growth. Fermentations were carried out in bioreactors (300 mL) at a controlled temperature of 30°C. We monitored substrate utilization and metabolite production over 72 h fermentation using HPLC-RI and ^1^H-NMR. In total, five independent fermentations were conducted (F1–F5).

The initial cell count was 7 log CFU/mL, and the cell count increased to 8.7 ± 0.1 log CFU/mL during fermentation. The highest increase in cell counts was observed during the first 24 h. The optical density increased most between 24 and 48 h (Table 2). Glucose was metabolized immediately, and the L-rhamnose levels were reduced to less than 5 mM after 48 h fermentation (Table 2). Acetate, lactate, and formate accumulated at levels of 2.5 ± 1.1 mM, 22.8 ± 11.1 mM, and 1.0 ± 0.7 mM during fermentation, respectively. 1,2-PD, propanol, propanal, and propionate were first detected after 24 h with maximum levels at 48 h for 1,2-PD and propanal and 72 h for others. After 48 h, the 1,2-PD level decreased in F1–F5 by ∆0.3–2.7 mM. Likewise, the amount of propanal was reduced by ∆0.0–1.9 mM. Based on HPLC-RI results, 2.4 ± 2 mM 1,2-PD, 1.1 ± 1 mM propanal, 3.1 ± 0.8 mM propanol, and 15.9 ± 3.4 mM propionate were detected after 72 h fermentation (Table 2). ^1^H-NMR analysis was performed of samples collected from F4 and F5, confirming the production of propionate (14.0 ± 1.9 mM) and the presence of lactate (23.8 ± 2.1 mM), formate (2.0 ± 0.5 mM), and acetate (3.3 ± 0.9 mM). Under the consideration that the homofermentative *L. coryniformis* produced about 2 mM lactate from glucose, the final ratio of lactate:propionate + propanol was 1.1 ± 0.1. Of the used L-rhamnose, 77.2% ± 5.4% (Cmol/Cmol) could be recovered as SCCA (lactate, acetate, propionate, and formate), propanal, propanol, and 1,2-PD. Taken together, *L. coryniformis* metabolized L-rhamnose to almost equimolar levels of propionate + propanol and lactate while concurrently producing low levels of acetate and formate.

**TABLE 2:** Cell concentrations, substrate utilization, and metabolites^*a*^

Time (h)	Cell count (CFU/mL)	Optical density (600 nm)	Concentration (mM)								
			L-rhamnose	D-glucose	Lactate	1,2-PD	Propionate	Propanol	Propanal	Acetate	Formate
0	7.0 ± 0.0^B^	0.3 ± 0.1^B^	32.0 ± 6.0^C^	1.2 ± 1.1^A^	0.0 ± 0.0^B^	0.0 ± 0.0^A^	0.0 ± 0.0^C^	0.0 ± 0.0^B^	0.0 ± 0.0^A^	0.0 ± 0.0^B^	0.0 ± 0.0^A^
24	8.4 ± 0.3^A^	0.7 ± 0.2^B^	22.4 ± 3.6^A^	0.4 ± 0.3^A^	9.5 ± 6.4^B^	2.3 ± 2.5^A^	1.5 ± 1.4^C^	0.5 ± 0.5^B^	0.7 ± 0.9^A^	0.5 ± 0.8^B^	0.3 ± 0.6^A^
48	8.6 ± 0.1^A^	1.8 ± 0.7^A^	3.4 ± 1.3^B^	0.4 ± 0.3^A^	25.3 ± 7.3^A^	2.9 ± 2.7^A^	9.7 ± 3.6^A^	2.2 ± 0.8^A^	1.1 ± 1.0^A^	0.8 ± 0.9^B^	0.1 ± 0.7^A^
72	8.7 ± 0.3^A^	1.8 ± 0.6^A^	2.9 ± 1.6^B^	0.4 ± 0.3^A^	22.8 ± 11.1^A^	1.5 ± 1.8^A^	16.3 ± 7.0^B^	2.9 ± 1.3^A^	0.7 ± 0.9^A^	2.2 ± 1.2^A^	0.6 ± 0.7^A^

^*a*^ Cell concentration and optical density of *L. coryniformis* were measured during growth on L-rhamnose and D-glucose in a bioreactor at 30°C for 72 h. Substrate and metabolite concentrations were analyzed using HPLC-RI. The fermentation was conducted in five biological replicates (*n* = 5). Different uppercase superscript letters indicate significant differences between groups within each column at different time points determined with Tukey’s test (*P* < 0.05). Data are shown as mean ± SD.

### Genes related to deoxyhexose metabolism and expression profiles of *L. coryniformis* during L-rhamnose utilization

In *L. coryniformis* DSM 20001, genes related to L-rhamnose utilization and 1,2-PD metabolism were located on two gene clusters (LC20001_RS04725 to LC20001_RS04750 and (LC20001_RS04205 to LC20001_RS04315), respectively (Fig. 1). The *rha* operon contained genes encoding transcriptional regulator (Gene ID: 65916553), MFS transporter (65916554), and *rhaM* (65916556)*, rhaA* (65916557), *rhaB* (65916555), and *rhaD* (65916558) encoding enzymes responsible for converting L-rhamnose into DHAP and L-lactaldehyde. The *pdu* operon of *L. coryniformis* harbored genes related to propanediol utilization and also encoded the structural proteins that form the microcompartment housing (BMC) similar to *L. reuteri* DSM 20016 (16).

To investigate gene regulation during growth in the presence of L-rhamnose, expression levels of key genes associated with L-rhamnose and 1,2-PD metabolism (Table 1) were determined from samples collected at four different fermentation stages of F4 and F5. RNA was isolated and reverse transcribed, and gene expression was determined using qPCR. Gene expression levels were calculated relative to three housekeeping genes *gyrB*, *gap*, and *pheS*. We also calculated expressions relative to the transcription levels of the 16S rRNA gene. Expression levels at 6 h were used as a reference calculator.

**Fig 2:**
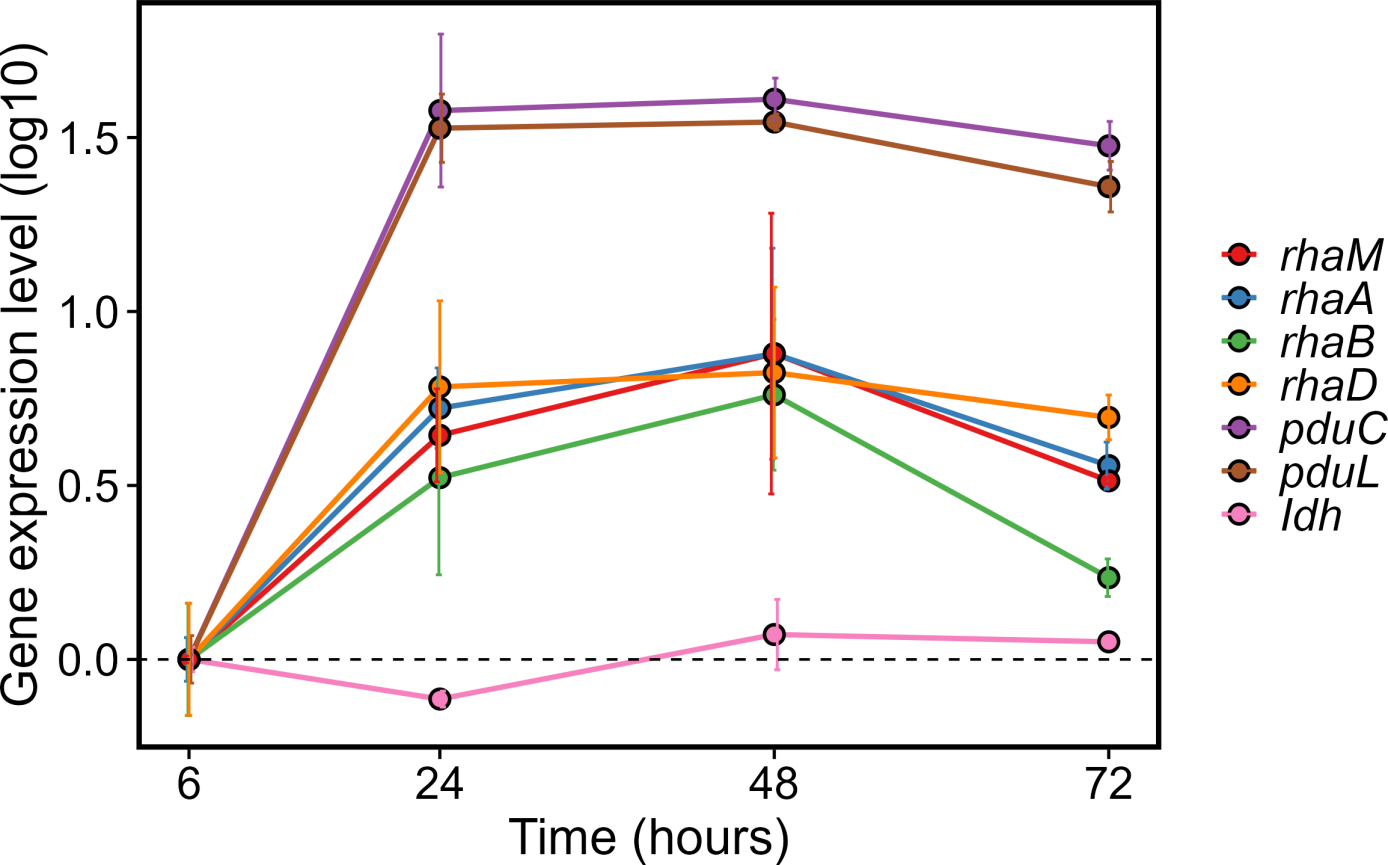
Relative gene expression levels of L-rhamnose and 1,2-PD metabolism-related genes. The expression of L-rhamnose and 1,2-PD utilization-related genes in samples collected at different time points throughout the 72 h fermentation was determined using qPCR. *gap* was used as housekeeping gene, and data were normalized based on expression at 6 h. Data are shown as log expression levels.

When the *gap* was used as a housekeeping gene, all tested genes associated with L-rhamnose metabolism were upregulated: *rhaM* (ninefold), *rhaA* (eightfold), *rhaB* (sixfold), and *rhaD* (sevenfold) at 48 h compared with 6 h. Genes related to 1,2-PD utilization (*pduC* and *pduL*) were overexpressed 40-fold (*pduC*) and 34-fold (*pduL*) at 24 h and 48 h. For *ldh*, a 0.1–0.2-fold downregulation was observed at 48 and 72 h (Fig. 2). In general, gene expression patterns were similar when *gap* (Fig. 2), *gyrB* (Fig. S1A), and *pheS* (Fig. S1B) were used as housekeeping genes with few exceptions. L-rhamnose metabolism-related genes were all downregulated at 72 h when *gyrB* was used as the housekeeping gene (Fig. S1A), and relative expression levels were, in general, higher when *pheS* was used as the housekeeping gene (Fig. S1B).

When the expression level of target genes was calculated relative to the 16S rRNA gene, the relative expression of *rhaA*, *rhaM*, *rhaB*, and *rhaD* was 1.1–2.1-fold at 24 and 48 h and 0.0–0.1-fold at 72 h compared with 6 h. Relative expression of *pduC* was highest at 24 h (13-fold expression) relative to the 16S rRNA gene and was 7-fold and 0.2-fold at 48 and 72 h, respectively (Fig. S1C). In summary, the expression levels of gene-transcribing enzymes related to the metabolism of the intermediate 1,2-PD were higher than those of L-rhamnose utilization-related genes.

### Inhibitory activity of individual SCCA against indicator microorganisms

*L. coryniformis* fermentates were characterized by the presence of multiple SCCA. With the aim of determining the potential antimicrobial activity of the fermentates, we first analyzed the inhibitory effect of individual SCCA, namely, lactate, propionate, formate, and acetate. We performed a twofold dilution assay and used selected individual strains of Gram-negative *Enterobacteriaceae* (*E. coli* DSM 19206, *K. oxytoca* DSM 5115, and *S. enterica* CCM 4420), the Gram-positive *Enterobacteriaceae* (*S. aureus* DSM 1104), the yeasts (*C. albicans* DSM 10697, *P. kluyveri* FMT 3000, and *S. cerevisiae* FMT 3003), and the molds (*A. niger*, *P. roqueforti*, and *P. purpurogenum*) as indicator organisms. *E. coli* DSM 19206 (serotype O157:H7) is a pathogen from contaminated water and undercooked beef (18). *S. aureus* has been associated with raw dairy products and can produce enterotoxins (19). *K. oxytoca* is found in ready-to-eat foods, posing a risk to consumers (20). *C. albicans*, a nosocomial pathogen, can also contaminate food (21). *A. niger* and *P. roqueforti* cause spoilage in bread (22), while *P. purpurogenum* is linked to fruit spoilage (23). All tested strains grew at pH 4.5, with the exception of *S. aureus*, which was tested at pH 5.5.

The MIC_50_ values of propionate, acetate, and formate against *Enterobacteriaceae* were similar, ranging from 1.6 to 2.6 mM for *E. coli*, 0.9 to 1.2 mM for *S. enterica*, and 3.3 to 5.2 mM for *K. oxytoca*. A considerably higher MIC_50_ value of lactate was observed for *K. oxytoca* (42.9 ± 1.7 mM) than for *E. coli* (15.1 ± 6 mM) and *S. enterica* (6.6 ± 1.9 mM) (Table 3). At pH 5.5, propionate (16.3 ± 1.8 mM) was the strongest SCCA against *S. aureus* followed by acetate (20.0 ± 4.9 mM) and formate (44.2 ± 7.4 mM). *S. aureus* was not inhibited in the presence of 50 mM lactate. Among the yeast indicators, only propionate inhibited *C. albicans* (12.6 ± 5 mM), while SCCA did not affect the growth of *P. kluyveri* and *S. cerevisiae* at levels of up to 50 mM. Propionate completely inhibited *P. roqueforti* (MIC 50 mM), *A. niger* (MIC 25 mM), and *P. purpurogenum* (MIC 12.5 mM) while reducing the growth of molds at lower concentrations (6.25–12.5 mM) (Fig. S2). Acetate inhibited the growth of *A. niger* (MIC 50 mM) and *P. purpurogenum* (MIC 25 mM) (Fig. S2A and C). Growth reduction was observed for *P. roqueforti* at 25–50 mM (Table 3). The presence of formate at 25–50 mM reduced mold growth, whereas lactate did not affect the growth of the tested molds (Fig. S2). In the presence of propionate, spore formation of *A. niger* during the 7-day incubation period was delayed compared to controls (Fig. S4).

**TABLE 3:** Antimicrobial activity of SCCAs against indicator strains**^*a*^**

SCCA	MIC_50_ (mM)									
	*E. coli*	*S. enterica*	*K. oxytoca*	*S. aureus*	*C. albicans*	*P. kluyveri*	*S. cerevisiae*	*A. niger^b^*	*P. roqueforti^b^*	*P. purpurogenum^b^*
Lactate	15.1 ± 6.0^aA^	6.6 ± 1.9^aA^	42.9 ± 1.7^aB^	>50	>50	>50	>50	>50	>50	>50
Formate	1.6 ± 0.6^bB^	1.2 ± 0.1^bB^	3.3 ± 0.2^bB^	44.2 ± 7.4^aA^	>50	>50	>50	50	25–50	25–50
Acetate	1.9 ± 0.8^bB^	0.9 ± 0.2^bB^	5.2 ± 0.3^bB^	20.0 ± 4.9^bA^	>50	>50	>50	25–50	25–50	12.5–50
Propionate	2.6 ± 1.2^bB^	1.0 ± 0.2^bB^	4.8 ± 0.6^bB^	16.3 ± 1.8^bA^	12.6 ± 5.0^A^	>50	>50	6.2–50	12.5–50	6.2–50

^*a*^ The minimal inhibitory concentrations of lactate, formate, acetate, and propionate to reduce cell density of bacterial and yeast indicator to 50% (MIC_50_) and the concentration ranges to reduce and inhibit mold growth were determined using twofold dilution assays at pH 4.5, except for *S. aureus*, which was tested at pH 5.5. Different lowercase superscript letters indicate differences within the column of the microorganism by Tukey’s test (*P* < 0.05). Different uppercase superscript letters indicate differences within the row of the SCCA by Tukey’s test (*P* < 0.05). Data are shown as mean ± SD (*n* ≥ 3).

^*b*^ Based on duplicates.

Taken together, propionate was the most efficient growth inhibitor among the four tested SCCA against the tested bacterial and mold indicator strains while the tested yeast showed higher resistance.

### Antimicrobial efficiency of fermentates and contribution of individual SCCA to inhibition

Next, we examined the inhibitory potential of fermentates F1–F4 using a twofold broth dilution assay. To determine the contribution of individual SCCA to fermentate antimicrobial activity, we additionally prepared synthetic SCCA mixtures (sSCCA) that lacked propionate, lactate, or acetate. Bacterial and mold indicators were tested, and only *C. albicans* was included in the assay as the most sensitive yeast toward the antimicrobial activity of SCCA. Growth inhibition was calculated as the dilution factor of the fermentate that reduced the cell density of indicator strains to 50% (DF_50_) compared with controls grown without fermentate. Only F4 was tested for the inhibition of mold indicators.

F1 contained the highest total concentration of SCCA (57 mM), resulting in higher DF_50_ values for all the bacterial indicator strains than F3, which had the lowest overall SCCA levels (29 mM). For example, the DF_50_ value for the inhibition of *E. coli* was 9.1 ± 3.1 mM for F1 and 3.5 ± 0.1 mM for F3 (Table 4). Compared with *E. coli* and *K. oxytoca*, *S. enterica* was the most sensitive to the inhibitory activity of fermentates with 1.4–3.6-fold higher DF_50_ values. For *E. coli*, the DF_50_ values of fermentates and sSCCA were within the same range, while the DF_50_ values of sSCCA were significantly higher than for fermentates for *S. enterica* and *K. oxytoca*, except for F4 (Table 4).

**TABLE 4:** Antimicrobial activity of fermentates and sSCCA mixtures^*a*^

Fermentation	Fermentate or sSCCA mixtures	Metabolites in fermentates and sSCCA (mM)				Dilution fold to reduce cell density of indicators to 50% (DF_50_)^*c*^							
		Lactate	Formate	Acetate	Propionate	*E. coli*	*S. enterica*	*K. oxytoca*	*S. aureus*	*C. albicans*	*A. niger^b^*	*P. roqueforti^b^*	*P. purpurogenum^b^*
F1	Fermentate	32	2	4	19	9.1 ± 3.1^A^^*b*^	17.4 ± 5.2^AB^	6.1 ± 1.2^AB^	NT	NI	NT	NT	NT
	sSCCA	32	2	4	19	9.4 ± 2.4^A^	24.6 ± 6.3^A^	6.4 ± 0.6^A^	NI	NI	NT	NT	NT
	sSCCA-P	32	2	4	0	3.6 ± 0.3^B^	9.0 ± 1.5^B^	2.0 ± 0.0^C^	NI	NI	NT	NT	NT
	sSCCA-L	0	2	4	19	8.0 ± 1.4^AB^	20.5 ± 4.1^A^	4.5 ± 0.5^B^	NI	NI	NT	NT	NT
	sSCCA-A	32	2	0	19	7.8 ± 1.2^AB^	17.6 ± 1.1^AB^	4.6 ± 0.6^B^	NI	NI	NT	NT	NT
F2	Fermentate	19	1	2	17	6.2 ± 1.0^A^^*b*^	8.4 ± 2.0^CD^	3.2 ± 0.2^B^	NT	NI	NT	NT	NT
	sSCCA	19	1	2	17	5.8 ± 2.0^A^	20.4 ± 2.6^A^	4.7 ± 0.8^A^	NI	NI	NT	NT	NT
	sSCCA-P	19	1	2	0	NI	4.3 ± 0.7^D^	NI	NI	NI	NT	NT	NT
	sSCCA-L	0	1	2	17	4.9 ± 2.8^A^	18.0 ± 3.7^AB^	3.5 ± 0.2^B^	NI	NI	NT	NT	NT
	sSCCA-A	19	1	0	17	5.2 ± 2.0^A^	13.9 ± 1.2^BC^	3.5 ± 0.2^AB^	NI	NI	NT	NT	NT
F3	Fermentate	14	1	2	12	3.5 ± 0.1^A^^*b*^	3.9 ± 1.7^B^	NI	NT	NI	NT	NT	NT
	sSCCA	14	1	2	12	3.4 ± 2.9^A^	12.8 ± 2.7^A^	3.3 ± 0.1^A^	NI	NI	NT	NT	NT
	sSCCA-P	14	1	2	0	NI	3.2 ± 0.2^B^	NI	NI	NI	NT	NT	NT
	sSCCA-L	0	1	2	12	3.7 ± 1.2^A^	12.1 ± 1.7^A^	2.5 ± 0.3^A^	NI	NI	NT	NT	NT
	sSCCA-A	14	1	0	12	3.7 ± 1.4^A^	10.1 ± 0.4^A^	2.7 ± 0.5^A^	NI	NI	NT	NT	NT
F4	Fermentate	25	0	2	18	12.0 ± 0.7^A^^*b*^	25.3 ± 6.3^A^	7.1 ± 0.4^A^	NT	NI	2.0 ± 0.0	2.0 ± 0.0	2.0 ± 0.0
	sSCCA	25	0	2	18	8.1 ± 0.7^B^	26.7 ± 3.3^A^	6.2 ± 1.2^A^	NI	NI	4.0 ± 0.0	4.0 ± 0.0	4.0 ± 0.0
	sSCCA-P	25	0	2	0	NI	8.8 ± 2.1^B^	NI	NI	NI	NI	NI	NI
	sSCCA-L	0	0	2	18	8.3 ± 1.1^B^	30.8 ± 6.2^A^	5.7 ± 1.9^A^	NI	NI	4.0 ± 0.0	4.0 ± 0.0	6.0 ± 0.0
	sSCCA-A	25	0	0	18	7.1 ± 0.0^B^	22.1 ± 3.5^A^	4.7 ± 1.1^A^	NI	NI	4.0 ± 0.0	4.0 ± 0.0	4.0 ± 0.0

^*a*^ Antimicrobial activity of fermentates, sSCCA, and sSCCA without propionate, lactate, or acetate was determined using twofold broth dilution assays. Dilution folds indicate the dilutions of fermentates and sSCCA mixtures that reduce the cell density of bacterial and fungal indicators to 50% (DF_50_). The concentrations of lactate (L), formate (F), acetate (A), and propionate (P) in SCCA-mimicked levels present in the fermentates. Different uppercase superscript letters indicate differences for each replicate within the column by Tukey’s test (*P* < 0.05). Data are shown as mean ± SD (*n* ≥ 3).

^*b*^ Based on duplicates.

^*c*^ NT, not tested; NI, no inhibition.

To determine how individual SCCA contributed to the antimicrobial activity of fermentates, we compared DF_50_ values of sSCCA with SCCA-A, sSCCA-P, and sSCCA-L, which lacked acetate, propionate, and lactate in the mixtures, respectively. The inhibitory activity of sSCCA-P toward *E. coli*, *K. oxytoca*, and *S. enterica* was significantly lower than that of sSCCA-L and sSCCA-A, and *K. oxytoca* showed lower sensitivity to sSCCA mixtures compared with *E. coli*.

*C. albicans* and *S. aureus* were not inhibited by fermentates and sSCCA mixtures (Table 4). The F4 fermentate and the corresponding sSCCA mixtures reduced the growth of indicator molds except sSCCA-P. Extensive mycelia formation was observed during growth with sSCCA-P indicating the relevance of propionate on the inhibitory activity of sSCCA mixtures (Fig. S2A through C).

Taken together, our results indicate that *L. coryniformis* fermentates were suitable antimicrobials against *Enterobacteriaceae* and molds at pH 4.5, mainly due to the activity of propionate.

### Synergistic activities between SCCA present in fermentates

In *L. coryniformis* fermentates, the SCCA propionate, lactate, acetate, and formate were detected. To explore the possibility of synergistic antimicrobial activities of SCCAs in fermentates and sSCCA mixtures, we related the actual SCCA concentrations at the DF_50_ value to the MIC_50_ values obtained for individual SCCA for *E. coli*, *S. cerevisiae*, and *K. oxytoca* and the molds, which were the most susceptible.

For *Enterobacteriaceae* and molds, the levels of acetate, lactate, and formate present in fermentates and sSCCA were 0.02–0.3-fold lower than the MIC_50_ values of the individual compounds (Table 5). In contrast, levels of propionate were 0.4–1.7-fold of the MIC_50_ for bacteria and the values with reduced growth for the molds (Table 5), suggesting low synergistic antimicrobial activity of the SCCA present in the fermentates and sSCCA, whose preservative effect was mainly due to propionate.

**TABLE 5:** Contribution of individual SCCA in fermentates/sSCCA to antimicrobial activity against *Enterobacteriaceae* and molds^*a*^

Indicator strains	Fermentate or sSCCA	Ratio levels of SCCA in fermentate or sSCCA to MIC_50_			
		Lactate	Formate	Acetate	Propionate
*E. coli*	Fermentate	0.2 ± 0.1	0.1 ± 0.1	0.2 ± 0.1	0.9 ± 0.3
	sSCCA	0.2 ± 0.0	0.1 ± 0.1	0.1 ± 0.0	0.8 ± 0.2
*S. enterica*	Fermentate	0.3 ± 0.2	0.1 ± 0.1	0.3 ± 0.2	1.7 ± 1.1
	sSCCA	0.1 ± 0.0	0.1 ± 0.0	0.1 ± 0.0	0.6 ± 0.0
*K. oxytoca*	Fermentate	0.1 ± 0.1	0.1 ± 0.1	0.1 ± 0.1	1.0 ± 0.6
	sSCCA	0.1 ± 0.0	0.1 ± 0.0	0.1 ± 0.0	1.0 ± 0.0
*A. niger^b^*	Fermentate	NI^*c*^	0.0 ± 0.0	0.04 ± 0.0	1.4 ± 0.0
	sSCCA	NI	0.0 ± 0.0	0.02 ± 0.0	0.7 ± 0.0
*P. roqueforti^b^*	Fermentate	NI	0.0 ± 0.0	0.04 ± 0.0	0.7 ± 0.0
	sSCCA	NI	0.0 ± 0.0	0.02 ± 0.0	0.4 ± 0.0
*P. purpurogenum^b^*	Fermentate	NI	0.0 ± 0.0	0.08 ± 0.0	1.4 ± 0.0
	sSCCA	NI	0.0 ± 0.0	0.04 ± 0.0	0.7 ± 0.0

^*a*^ Actual concentrations of SCCA at DF_50_ values were related to the MIC_50_ of the individual SCCA to determine the contribution to the antimicrobial activity of the mixtures against *E. coli*, *S. enterica*, *K. oxytoca*, *A. niger*, *P. roqueforti*, and *P. purpurogenum*. The data are shown as mean ± SD (*n* ≥ 3).

^*b*^ Based on duplicates.

^*c*^ NI, no inhibition.

## DISCUSSION

### *L. coryniformis* efficiently metabolized L-rhamnose to propionate

L-rhamnose utilization to propionate by *L. coryniformis* was first mentioned by Buljubašić et al. (7). We have optimized the small-scale batch fermentation for *L. coryniformis*, which resulted in higher propionate production levels. In this study, fermentation was run in batch mode at a temperature of 30°C resulting in the utilization of 90.6% of the supplied L-rhamnose and a sixfold increase in propionate production compared with fermentations conducted at 37°C highlighting the critical impact of temperature on both L-rhamnose utilization and propionate synthesis by *L. coryniformis*.

L-rhamnose metabolism-related genes (*rhaM*, *rhaA*, *rhaB*, and *rhaD*) were upregulated compared with the control (6 h fermentation), while L-rhamnose was present at levels > 3.5 mM. Similarly, L-fucose utilization encoding genes were upregulated during the growth of *Roseburia inulinivorans* on L-fucose when compared with the growth on D-glucose (24), indicating a regulatory effect of substrate availability on the transcription of the *rha* operon. During L-rhamnose metabolism, only low amounts of 1,2-PD accumulated during fermentation due to simultaneous conversion to propanal, propanol, and propionate initiated by cobalamin-dependent glycerol/diol dehydratase (pduCDE) (13, 16, 25).

In agreement, genes related to 1,2-PD utilization were highly upregulated during the growth of *L. coryniformis* on L-rhamnose. Moreover, during the L-rhamnose conversion in *Clostridium phytofermentans*, expression of *pduCDE* and *pduL* was higher (32-fold) compared with *rhaB* (8.5-fold), *rhaA* (11.1-fold), and *rhaM* (2-fold) (15) similar to our results. Likewise, the BMC proteins of *Clostridium beijerinckii* DSM 6423 were significantly upregulated (9- to 12-fold compared with D-glucose-grown cells) during growth on L-rhamnose (11).

In general, relative expression levels of target genes were higher when related to protein-encoding mRNA transcripts than to expression levels of ribosome-related transcripts, i.e., expression of the 16S rRNA gene. This is consistent with observations in *Staphylococcus epidermidis* and *E. coli* that demonstrated that ribosomal content increased during bacterial exponential growth before protein synthesis (26).

### L-rhamnose metabolism yielded mainly lactate and propionate

L-rhamnose fermentation by *L. coryniformis* yielded two primary metabolites, lactate, and propionate, in nearly equimolar amounts.

In *E. coli*, L-lactaldehyde was catabolized by FucO to 1,2-PD, which is excreted into the environment as a terminal electron acceptor at anaerobic conditions (27). In *Lacticaseibacillus rhamnosus* GG, L-lactaldehyde is likely metabolized to 1,2-PD through the action of two enzymes, aldehyde-alcohol dehydrogenase and iron-containing alcohol dehydrogenase (8). *L. coryniformis* lacks a FucO homolog but possesses a gene encoding a protein (LC20001_RS09775) with 61% identity to the aldehyde-alcohol dehydrogenase of *L. rhamnosus*.

In contrast, no homolog to lactaldehyde dehydrogenase (transforms lactaldehyde to lactate) encoding gene (*aldA*) was detected in the genome of *L. coryniformis* DSM 20001 similar to *L. rhamnosus* GG (8). This suggests that lactate was not produced from lactaldehyde but was formed from DHAP. DHAP can feed into glycolysis and yield a variety of fermentation metabolites such as lactate, acetate, and formate. The primary fermentation products of *C. phytofermentans* during growth on L-rhamnose and L-fucose were acetate, lactate, and ethanol, besides propanol and propionate (15). L-fucose metabolism of *R. inulinivorans* produced butyrate in addition to propanol and propionate (24). Based on these observations, it is likely that *L. coryniformis* transformed the pyruvate that became available during L-rhamnose metabolism, to its major fermentation metabolite lactate in agreement with the expression of *ldh* during the fermentation.

When *L. diolivorans* and *L. reuteri* utilized 1,2-PD, they produced equimolar amounts of propionate and propanol (16, 25). In contrast, *L. coryniformis* predominantly produced propionate from L-rhamnose. During the metabolism of L-rhamnose, NAD+ generated from the production of 1,2-PD may be reduced to NADH during pyruvate production. Similarly, lactate production from pyruvate generates NAD+, which could be consumed in the production of propionate instead of propanol, as propanol formation requires NADH. Additionally, formate or acetate may be produced to regenerate ATP and maintain cofactor balance (28).

We observed a transient accumulation of low levels of propanal during the 72 h fermentation despite its known toxicity. Previous studies investigating 1,2-PD metabolism of *L. reuteri* and *L. diolivorans* also reported temporary detectable propanal levels; longer fermentation periods led to the depletion of propanal (16, 25).

### Antimicrobial activity of fermentates differed between indicator strains and was dependent on pH

We evaluated the antimicrobial activity of fermentates and SCCA against a collection of bacteria, yeast, and molds. Although this analysis provides valuable insights into the antimicrobial activity of SCCA, it is important to note that only one strain of each species was tested. While sensitivity might differ between individual strains of a species, our data indicate that bacterial and mold indicator strains were more sensitive to individual SCCA, fermentates, and sSCCA mixtures than yeasts. Similarly, in previous studies it was observed that yeasts were less sensitive to propionic acid and longer-chain fatty acids compared with mold spores like *A. niger* and *P. roqueforti* or bacterial indicators (29, 30). The lower sensitivity of yeasts to weak acids has been attributed to their capability to regulate the internal pH of the cell and to a system for exporting anions (31).

Our results indicate the importance of initial pH on the antimicrobial activity of SCCA-containing fermentates and sSCCA. *S. aureus* could only be tested at pH 5.5 because growth inhibition occurred at pH 4.5. The higher pH value (pH 5.5) altered the proportion of dissociation of propionate compared with pH 4.5 as based on the Henderson-Hasselbalch equation; the total undissociated acid concentration of sSCCA was 22.2 mM at pH 4.5, while it was 5.0 mM at pH 5.5, for example, in F1. Similarly, it was previously reported that the antiyeast activity of propionate was lower when the pH was raised from 4.0 to 6.0 (32).

### Propionate was the major antimicrobial in fermentates, and activity was affected by the presence of metabolizable fermentation intermediates

Our fermentates contained up to 16 mM of propionate along with other SCCA. Propionate was identified as the major contributor to antimicrobial activity despite the presence of high levels of lactate. Lactate causes the cytoplasm to become more acidic, ultimately leading to growth inhibition (33), but shows less antimicrobial activity in pH-adjusted conditions compared with propionate and acetate (32). We did not observe any synergistic activity of the propionate and lactate, and the concentration of acetate in *L. coryniformis* fermentates was possibly too low to contribute to antimicrobial activity.

In fermentates F1, F2, and F4, propionate levels were high enough to suppress the growth of *C. albicans* but it was not inhibited. The antimicrobial activity of propionate might have been counteracted by the presence of lactate, which certain yeast and molds can use as a carbon source. *C. albicans* has previously been reported to utilize lactate and acetate, and it has been suggested that it possesses an active transport mechanism for SCCA across its plasma membrane, particularly for lactate and acetate (34, 35). *A. niger* grew better in the presence of lactate compared with controls, particularly at low pH levels (36). Dagnas et al. (37) reported that the growth rate of molds increased with the addition of lactate (37).

Similarly, *Salmonella* might have profited from the presence of a fermentation intermediate. In contrast to sSCCA mixtures, fermentates contained 1,2-PD in addition to SCCA. Since *S. enterica* possesses a B12-dependent glycerol dehydratase and a *pdu* operon similar to *L. coryniformis* (38), *Salmonella* can utilize 1,2-PD (39). The lower DF_50_ values in fermentates than sSCCA might be due to the generalizability of the utilization of 1,2-PD by *Salmonella*. These observations indicate that the composition of L-rhamnose-derived fermentates is of critical relevance for application as biopreservatives.

### Concluding remarks

Our study succeeded in increasing propionate production of *L. coryniformis* from L-rhamnose through batch fermentation under controlled pH and temperature conditions. *L. coryniformis* efficiently metabolized L-rhamnose to nearly equal amounts of propionate and lactate without substantial accumulation of pathway intermediates in agreement with higher expression of genes encoding enzymes involved in 1,2-PD metabolism than genes associated with L-rhamnose utilization. The antimicrobial activity of fermentates was related to propionate content but could be reduced if indicators were able to use 1,2-PD or lactate.

## SUPPLEMENTAL MATERIAL

Supplemental figures aem.01613-24-s0001.docx Relative gene expression and antifungal activity.

ASM does not own the copyrights to Supplemental Material that may be linked to, or accessed through, an article. The authors have granted ASM a non-exclusive, world-wide license to publish the Supplemental Material files. Please contact the corresponding author directly for reuse.
