# Supplementary material for: Investigation on L-rhamnose metabolism of Loigolactobacillus coryniformis subsp. coryniformis DSM 20001 and its propionate-containing fermentates
Source: Appl Environ Microbiol. 2024 Dec 18;91(1):e01613-24. doi: 10.1128/aem.01613-24 (PMC11784251; doi:10.1128/aem.01613-24)
Supplement: Supplemental figures — Relative gene expression and antifungal activity. [file aem.01613-24-s0001.docx]

**Supplemental Information**

**Investigation on L-rhamnose metabolism of *Loigolactobacillus coryniformis* subsp. *coryniformis* DSM 20001 and its propionate-containing fermentates**

Mensure Elvan Gezer^1#^, Kathrine Gravlund Fønss ^1#^, Maria Florencia Bambace^1^, Angeliki Marietou^1^, Sanne Sandberg Overby^1^, Ulrik Sundekilde^2^, Clarissa Schwab^1*^

^#^authors contributed equally

*corresponding author: [schwab@bce.au.dk](mailto:schwab@bce.au.dk)

^1^Department of Biological and Chemical Engineering, Aarhus University, Aarhus, Denmark

^2^Department of Food Science, Aarhus University, Aarhus, Denmark

1. **gyrB**


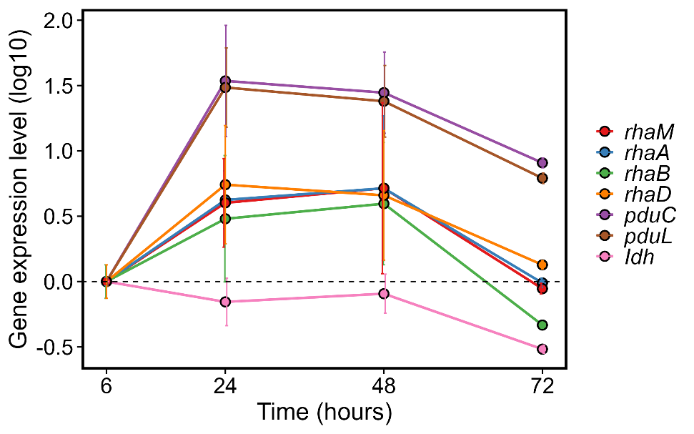


1. **pheS**


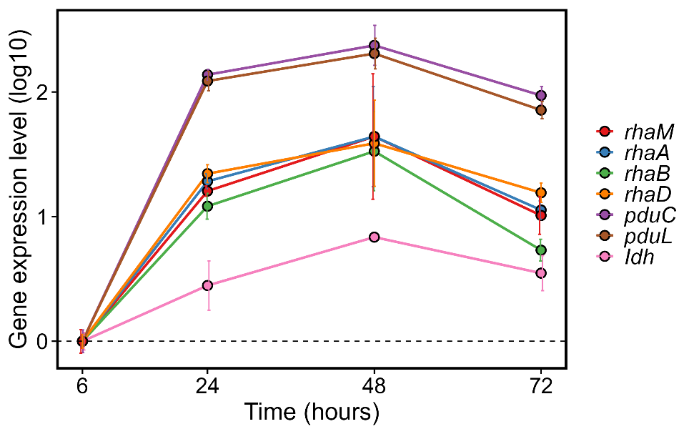


1. **16S rRNA**


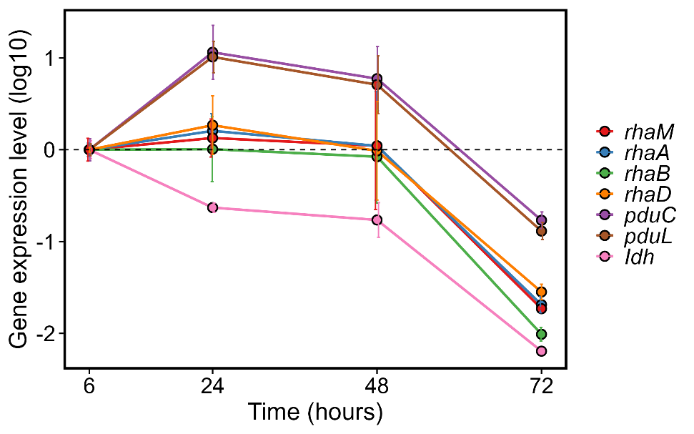


**Figure S1. Relative gene expression level of L-rhamnose and 1,2-PD metabolism-responsible genes**

The expression of L-rhamnose and 1,2-PD utilization-related genes in samples collected at different time points throughout the 72 h fermentation was detected using qPCR. *gyrB, pheS,* and *16S* rRNA genes were used as housekeeping genes, and data were normalized based on 6 h. Data are shown as log expression levels.

1. ***Aspergillus niger***


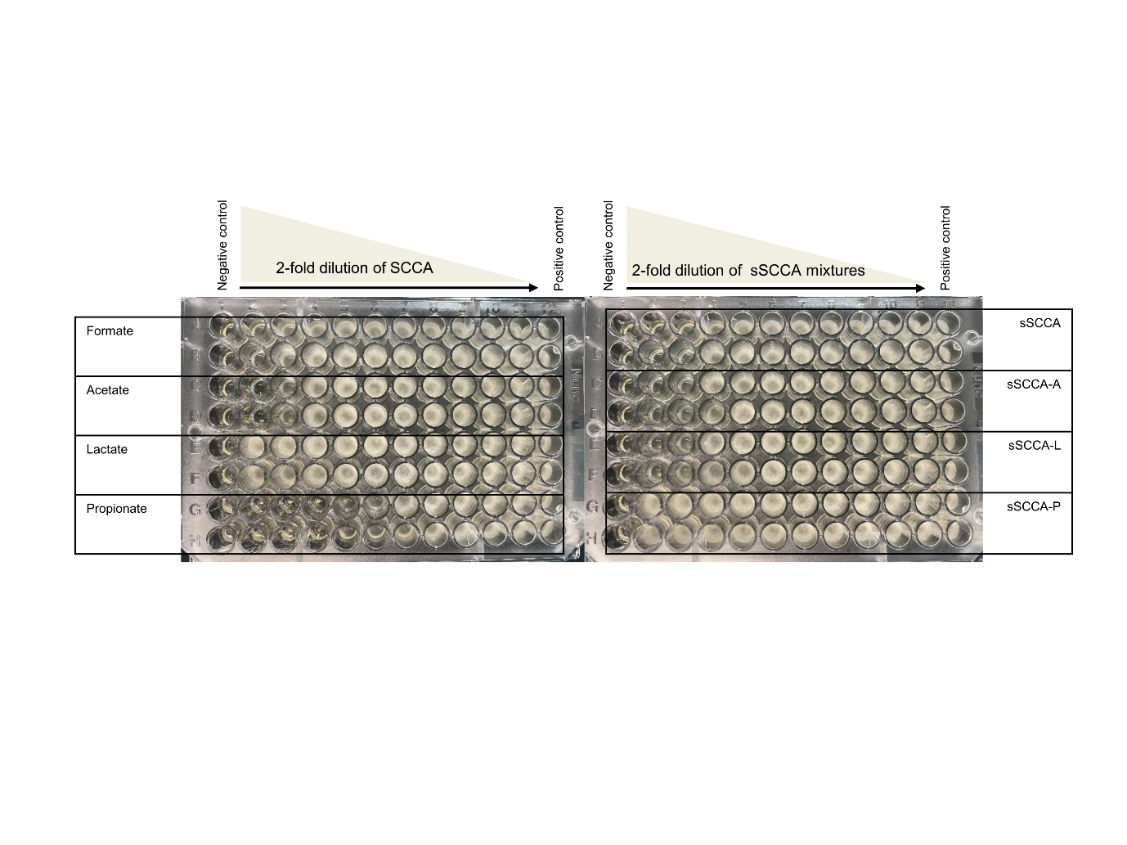


1. ***Penicillium roqueforti***


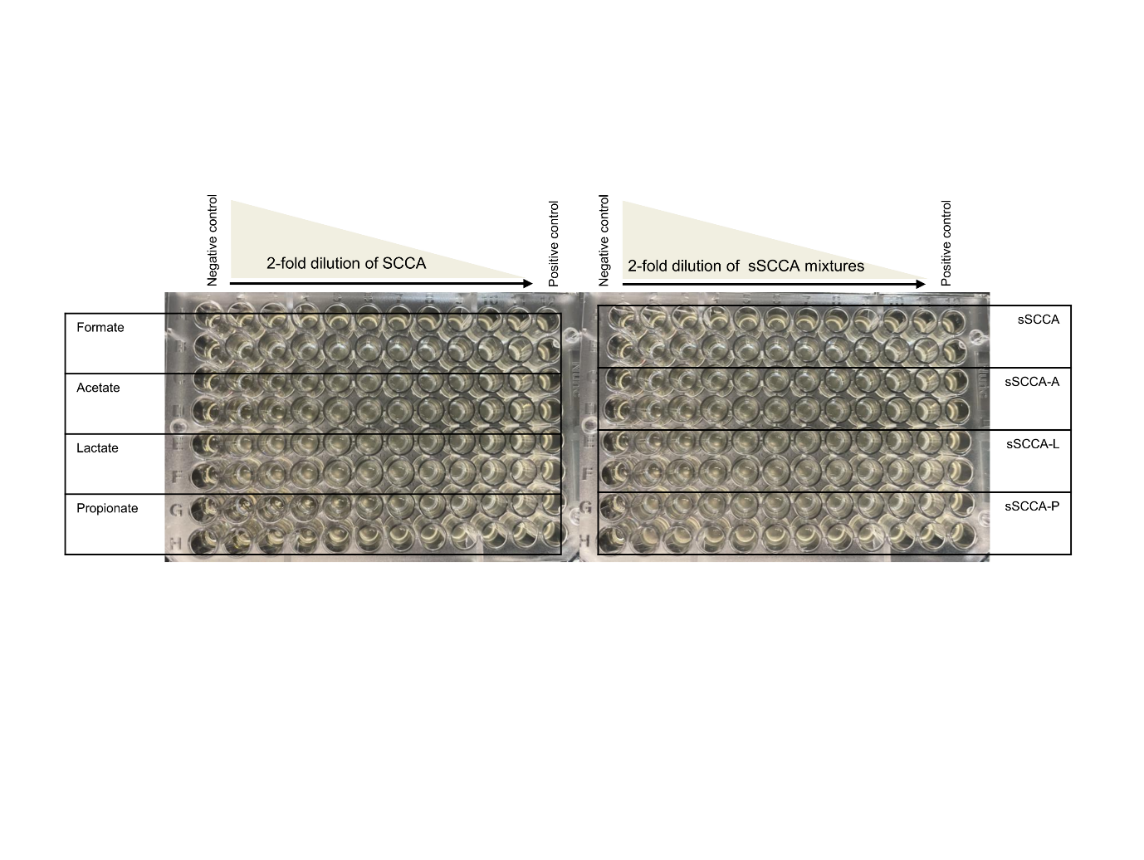


1. ***Penicillium purpurogenum***


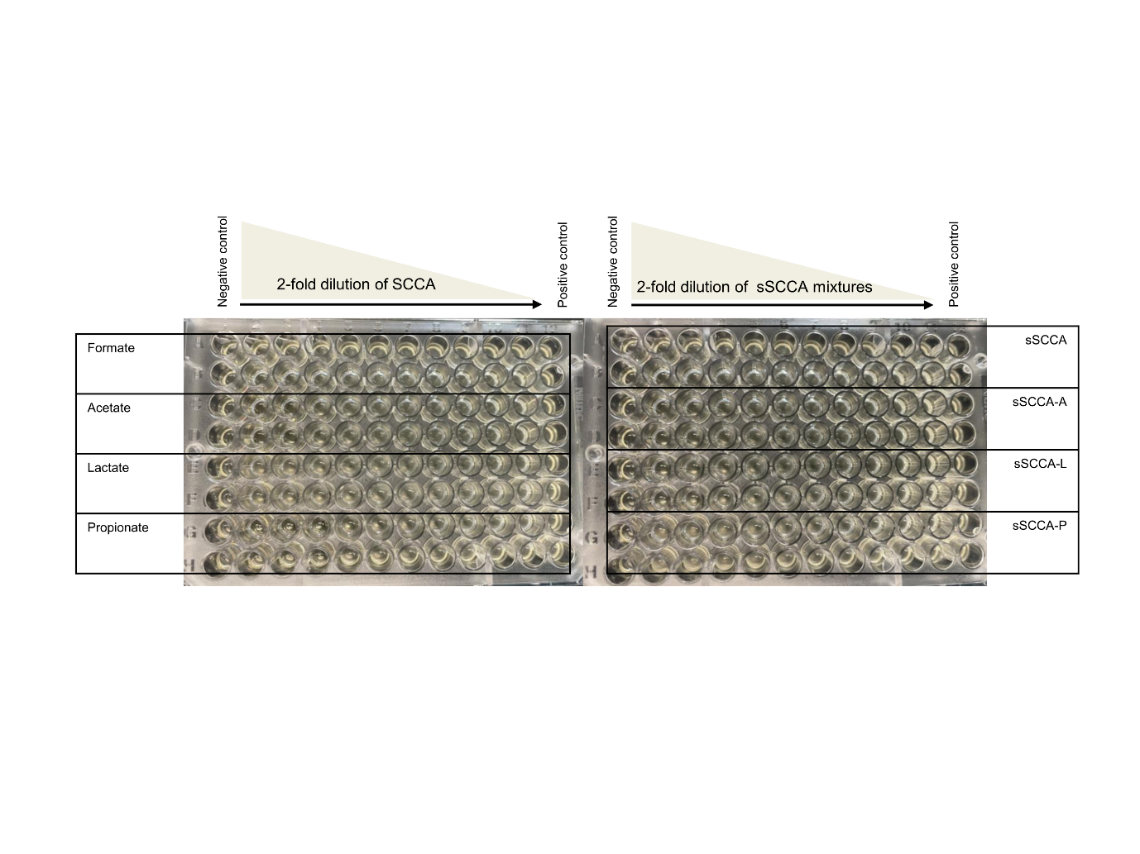


**Figure S2.** **Antimicrobial activity of SCCA and sSCCA mixtures against fungal strains.**

The minimal inhibitory concentrations of lactate, formate, acetate, and propionate were determined using two-fold dilution assays at pH 4.5. The pictures are representative of the results of the antifungal experiments.

**A**


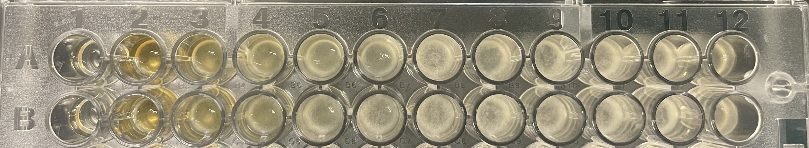


**B**


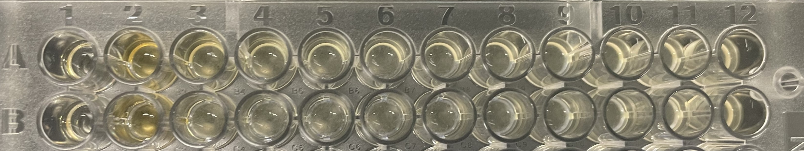


**C**


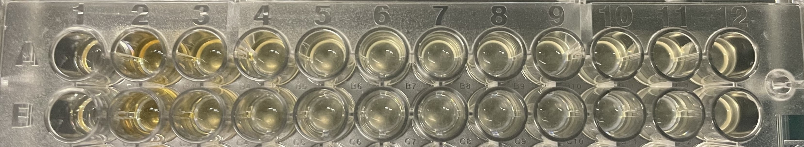


**Figure S3.** **Antimicrobial activity of fermentate against fungal strains (*A. niger* (A), *P. roqueforti* (B), and *P. purpurogenum* (C)).**

The minimal inhibitory concentration of fermentate was determined using two-fold dilution assays at pH 4.5. The pictures are representative of the results of the antifungal experiments.


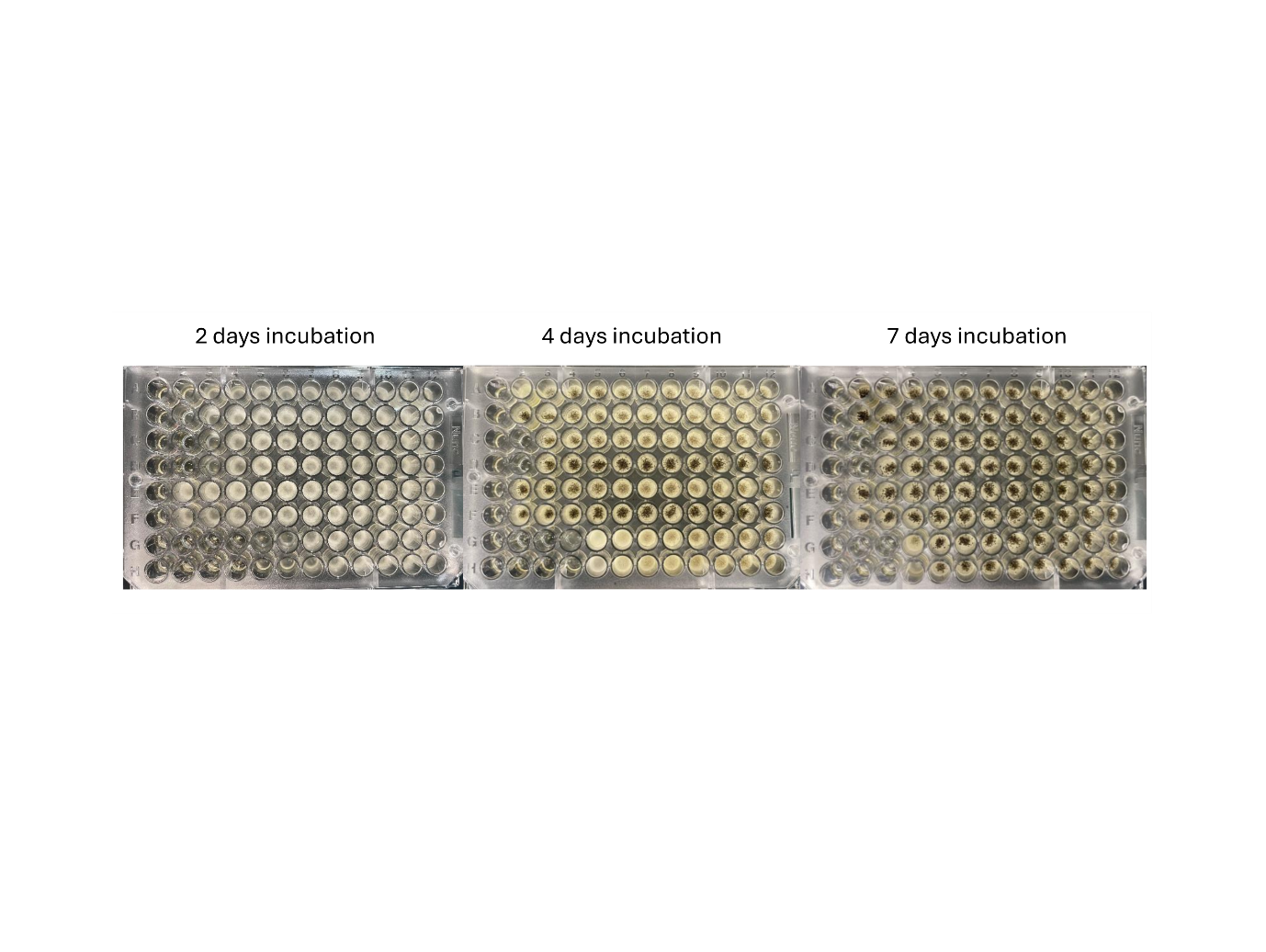


**Figure S4.** **Effect of the SCCA on spore formation of *A. niger*.**

The spore formation was observed during the 7-day incubation using two-fold dilution assays at pH 4.5. The pictures are representative of the results of the antifungal experiments.
